# Online videos: The hidden curriculum

**DOI:** 10.1111/eje.12766

**Published:** 2022-02-11

**Authors:** Marco Antonio Dias da Silva, Andresa Costa Pereira, Sibylle Vital, Rodrigo Mariño, Aghareed Ghanim, Mary Caroline Skelton‐Macedo, Argyro Kavadella, Afrodite Kakaboura, Sergio E. Uribe, Ilona Johnson, Domenico Dalessandri, Anthony Damien Walmsley

**Affiliations:** ^1^ University of Birmingham Birmingham UK; ^2^ Universidade Federal de Campina Grande Campina Grande Brazil; ^3^ University of Paris Paris France; ^4^ University of Melbourne Melbourne Victoria Australia; ^5^ Universidade de Sao Paulo Sao Paulo Brazil; ^6^ European University Cyprus Nicosia Cyprus; ^7^ National and Kapodistrian University of Athens Athens Greece; ^8^ Universidad Austral de Chile Valdivia Chile; ^9^ Riga Stradins University Riga Latvia; ^10^ Riga Technical University Riga Latvia; ^11^ Cardiff University Cardiff UK; ^12^ University of Brescia Brescia Italy

**Keywords:** dentistry, e‐learning/computers, independent, information handling, undergraduate

## Abstract

**Introduction:**

Dental undergraduates will access the Internet searching for learning materials to complement their training; however, open access content is not generally recommended by dental schools. This study aimed to evaluate how dental students are using online video content.

**Materials and Methods:**

Students from eight Universities (Athens, Birmingham, Brescia, Cardiff, Melbourne, Paris, Sao Paulo and Valdivia) representing three continents were invited to complete a survey on their access and learning from online videos.

**Results:**

International students behave similarly when studying dental content online. Of 515 respondents, 94.6% use the Internet as a learning tool. It was observed that videos are not frequently recommended during didactic lectures (9.6%). But many students (79.9%) will use YouTube for their learning which includes clinical procedures. Students will check online content before performing procedures for the first time (74.8%), to understand what was explained in class (65.9%) or read in books (59.5%), to relearn clinical techniques (64.7%) and to visualise rare procedures (49.8%). More than half of the students do not fully trust the accuracy or the reliability of online content. This does not prevent students from watching and sharing dental videos with classmates (64.4%). The content watched is not shared with teachers (23.3%) even when it contradicts what was learnt in the school (38.2%).

**Conclusion:**

This study concludes that students regularly integrate open access digital resources into learning portfolios but are hesitant to inform their teachers about their viewing habits. Students wish to receive critical skills on how to evaluate the material they encounter outside their traditional learning space.

## 1 INTRODUCTION

We are living in changing times where information can be accessed and created by everyone. The expansion of Internet access and the growing number of information and communication technologies (ICT), such as mobile devices, are responsible for promoting deep changes in the learning and teaching process.^1^ The use of technology is now commonplace within learning environments.^2^, ^3^ Students are likely to access online content to complement their learning before or after a traditional lecture.^1^, ^4^

When students are searching for instructional content, Google is chosen as the default search engine over other search engines for most searches.^5^ However, the ease of using the Internet brings new challenges and these are related to the quality, validity and reliability of the content.^6^, ^7^ Online content can be created and published by anyone without any peer reviews.^8^, ^9^

Infodemic is the term used by the World Health Organization to define the far‐reaching, spreading overabundance of information, including false or misleading materials, that makes it hard for people to find reliable online content.^10^, ^11^

The scientific judgement of an undergraduate student may not be able to critically review online content sensibly. A solution is for peer review content to be created by their teachers which leads to the delivery of high‐quality instructional online content.^9^, ^12^

The latest report from the Organisation for Economic Co‐operation and Development (OCDE), based on data from the Programme for International Student Assessment (PISA), shows that less than a half of digital natives 15‐year‐old is not able to accurately distinguish between fact or opinion on the Internet. It was also demonstrated that many pre‐undergraduates have difficulties in reading, exploring, interacting with online reading content as well as with task‐oriented navigation activities.^13^

For this generation, videos play a significant role in students' education.^13^ The use of images, text and videos leads to enhanced learning.^2^ Whilst video is a popular format, how they are designed and delivered may vary.^14^, ^15^ This also includes their use in flipped classrooms and video‐based training of clinical procedures.^16^, ^17^ Videos stimulate learning not only with traditional lectures but also with clinical training.^18^, ^19^ Such learning is a natural progression for students who are familiar with searching and watching videos online for personal and scholastic reasons.^20^

YouTube videos are considered as “How‐to” learning tools.^20^ Monthly over 2 billion logged‐in users visit YouTube^21^ and it has been observed that it is a source of health‐related information for 80% of its users.^22^ Videos are one of the most used and accessible electronic components and the content produced can be shared for free.^23^, ^24^, ^25^ Dental schools seem to ignore this reality and do not provide open online content. Most studies published in scientific journals concerning the use of videos in dental education focus on the offering of teacher‐created videos to their own students, under very specific and controlled situations.^26^, ^27^, ^28^, ^29^ As a result, online dental content is delivered by unreliable sources.^9^ Understanding how students use online dental educational videos during self‐learning sessions, out of the dental school, will be a major asset in integrating this behaviour into future methodological approaches. This study aimed to evaluate how undergraduate students are using online content as a complementary learning tool.

## 2 MATERIAL AND METHODS

### 2.1 Inclusion criteria

This international study compared the online behaviour of dental students from several schools in three continents when using Internet content as a complementary non‐curricular learning tool. As a part of a European Union‐funded project (number omitted), several dental schools were invited to collaborate. Eight schools, Athens, Birmingham, Brescia, Cardiff, Melbourne, Paris, Sao Paulo and Valdivia, participated in this study. Local coordinators organised recruitment, data collection and data input. School selection was partly due to previous research relationships and convenience. However, an additional rationale for country selection was related to their cultural background representation. For example, Australia and UK, despite their diverse ethnic composition,^15^ place responsibility on the individual and individual‐centred programmes, empowerment and personal enrichment. In contrast, countries like Chile are characterised by a more hierarchical societal structure, and a focus on the well‐being of the group rather than individuals.^16^ This would allow for the initial testing of the empirical hypothesis on the effect of the school environment in the use of online content as a complementary learning tool.

Dental students at each school were given an anonymous online survey. All students in the selected schools were invited to participate in this study. This study was reviewed and approved by the local University Ethical Committees at each University. Each respondent gave consent and had 4 weeks, after submitting the survey, to opt out of the research.

### 2.2 Questionnaire

An online self‐administered structured questionnaire was constructed by adapting and expanding previously published questionnaires.^1^, ^24^, ^30^, ^31^ The survey was delivered via Google drive using an account linked to a university email account. A short URL link to the survey was sent to the students, no personal data or email addresses were collected at any time. Each student was asked to create and keep a unique code in case they decided to opt out of the research.

The 24 questions were created in English, translated to Portuguese and Spanish and checked by native speakers. French, Italian and Greek dental schools opted to use the English version of the questionnaire. Closed‐ended, multiple‐choice questions were used. In some questions, the alternative “other” was offered. None of the questions was mandatory. The survey instrument was piloted, analysed and reviewed by invited dental teachers from different universities.

### 2.3 Data analysis

Jamovi,^32^ Bioestat^33^ and SPSS^34^ were used to analyse the data. Descriptive analysis was used on specific questions. Inferential analysis was conducted for identifying factors (age group, year of the dental course and location of the dental course) associated with students' usage of, and opinions on, online educational content. Parametric or non‐parametric tests (e.g. chi‐square or Fisher's exact test followed by odds ratio, Phi and Cramer for nominal, and Kruskal–Wallis Mantel–Haenszel for ordinal categories), as required, were used to identify the association between study variables and socio‐demographic and study characteristics.

## 3 RESULTS

In total, 515 students participated in the survey: Athens (*n* = 58), Birmingham (*n* = 23), Brescia (*n* = 15), Cardiff (*n* = 11), Paris (*n* = 138), Sao Paulo (*n* = 109), Melbourne (*n* = 112) and Valdivia (*n* = 49); the response rates varied from 3.0% to 30.5%. We received a similar number of answers from students of all years of the dental course. As expected, the majority of participants (79.5%) were 20‐ to 25‐years‐old with significant differences between locations (*p* < .01). Melbourne students were older than the others (*p* < .01; Table 1). By the year of study, in the Melbourne sample, most of the respondents were from the first 2 years of the course (*p* < .01).

**TABLE 1:** Dental students' characteristics and use of Internet content for dental education

Question	*n*	%
In which year of the Dental are you enrolled?		
1st	86	17.0
2nd	102	20.2
3rd	150	29.6
4th	77	15.2
5th	69	13.6
6th	22	4.4
How old are you?		
18–19 years	38	7.4
20–21 years	150	30.1
22–25 years	253	49.4
25+	67	13.1
Do you use online content as a complementary learning tool for your dental course studies?		
I always check for additional online content	142	27.6
When I have doubts	190	37.1
When I am studying for the assessments	104	20.2
Never	28	5.4
Only if it is demanded	28	5.2
Once a week	23	4.5
What is your preferred device to access dental education content online?		
Laptop computer	324	64.2
Smartphone	107	21.2
IPad/ computer tablet	46	9.1
Desktop computer	28	5.5
How did you learn about the dental educational video content?		
From searching the Internet	256	53.8
Recommendations from teaching staff	163	34.2
Recommendations from classmates	57	12.0
During the lectures, do your teachers recommend online videos as complementary content?		
Hardly ever	164	34.2
Occasionally	144	30.0
Sometimes	124	25.8
Frequently	33	6.9
Almost always	13	2.7
Never/Do not know	2	0.4
Which of the following top three sites do you use most to access dental subjects on your smartphone?		
Google	372	72.2
YouTube	288	55.9
University sites	225	43.7
PubMed	222	43.1
Wikipedia	149	28.9
Google Scholar	112	21.7
Search engines	43	8.3
Science direct	37	7.2
Personal sites	31	6.0
Organisations	30	5.8
Commercial sites	24	4.7
eCourse	19	3.7
Other	13	2.4
Have you ever learnt any dental course content from internet content?		
Yes. From videos	380	78.2
Yes. From Pictures (step by step)	273	56.2
Yes. From graphical illustration	138	28.4
Yes. From animations	134	27.6
Never	24	4.9
Where do you get the dental education videos from?		
YouTube	389	79.9
From my university's website or virtual learning environment	241	49.5
From other dental courses websites	148	30.4
From my friends	74	15.2
Other	18	3.6
Is there an official channel of your dental course on YouTube?		
Yes	49	10.2
Not aware	263	54.7
No	170	35.1
Have you ever learnt a clinical procedure from an online video?		
Yes	328	68.8
No	149	31.2
When it would be important to use an online video to better understand a clinical procedure?		
Before performing the procedure for the first time	362	74.8
To see what was explained in class	319	65.9
To remember how to perform	313	64.7
To see what you read in the book	288	59.5
To visualise the procedures rarely seen	241	49.8
After performing a procedure for the first time	85	17.6
In which cases would you use an internet video to learn from?		
All procedures	86	18.0
Most of the procedures	137	28.7
Some procedures	201	42.2
Few procedures	53	11.1
In your opinion what is the ideal length for an instructional video?		
1 min or less	5	1.0
Between 1 and 5 min	173	36.2
Between 5 and 15 min	256	53.6
Between 15 and 45 min	44	9.0
More than 45 min	1	0.2
What characteristics do you think a good dental education video should present?		
Voice over	340	70.2
Timestamps	311	64.3
Legends	247	51.0
Links for additional content	149	30.8
Music	72	14.9
Other	1	0.2
How useful do you think dental procedures videos on the school website would be?		
Extremely useful	328	68.0
Fairly useful	117	24.3
Average	26	5.4
Not very useful	8	1.7
Not at all useful	3	0.6
What part of the dental curriculum would you like to see dental procedures videos available on the dental course website?		
Clinical	318	85.5
Preclinical	278	74.7
Basic sciences	187	50.3
Research	107	28.8
Public engagement	78	21.0
In your opinion, which part of the dental curriculum works well with online videos?		
Clinical	325	67.0
Preclinical	300	61.9
Basic sciences	220	45.4
Have you ever shared/discussed an online video with a classmate?		
Yes	307	64.4
No	170	35.6
Have you ever shared/discussed an online video with a teacher?		
Yes	111	23.3
No	366	76.7
Overall, how confident are you with regard to the accuracy of the information on the internet?		
Noticeably confident	22	5.6
Fairly confident	169	43.0
Average	161	41.0
Not very confident	39	9.9
Not at all confident	2	0.5
Overall, how confident are you in the relevance of information on the internet to your dental course?		
Very confident	37	9.4
Fairly confident	171	43.6
Average	152	38.8
Not very confident	27	6.9
Not at all confident	5	1.3
Would you like to receive information about how to check the reliability of online content?		
Yes	303	77.9
No	86	22.1
What would you do if you watch an online video which contradicts what you learn from your dental course?		
Discuss with a classmate	194	40.8
Show to one of my teachers	181	38.2
Ignore the resource	79	16.6
Nothing	21	4.4

Note

Figures may not add due to missing values.

### 3.1 Finding video content

In all the participant dental courses, the large majority of dental students (94.5%) reported using Internet content as a complementary learning tool (Table 1). However, 5.3% only used when required, and another 4.45% reported reviewing Internet content once a week only. The most frequent reasons mentioned for using online content were clarification of facts (37.1%) and studying for the assessments (20.2%). About a quarter of respondents reported always checking for additional content (27.6%) (Table 1). Those who indicated using the Internet “Only when is required” or “Once a week” or “Never” were grouped into an “Infrequent users” group. Melbourne participants were more likely to be in the infrequent users' group than those from other schools (OR = 2.94; 95% CI: 1.34–6.39). It also appeared that for each year of study the likelihood to be infrequent users decreased (OR = 0.75; 95% CI: 0.68–0.83).

When students were asked about the preferred device to access dental education contents, computer (69.7%) was the most preferred, in particular laptops, followed by smartphones (21.2%) and tablet/iPad (9.1%; Table 1). The majority of students indicated that they learned about education contents online from searching the Internet (53.8%). Another 34.2% indicated that the source of such information was teaching staff and the remaining 12.0% reported that the source of information was other classmates. There were no significant differences by year of study. However, there were significant differences by country (*p* < .001). Students from Greece were more likely to have learned about online content from their teaching staff (OR = 3.41; 95% CI: 1.94–5.99).

In addition, when students were asked whether teaching staff recommend online videos as complementary content for their lectures, the majority (64.2%) responded “Never,” “Hardly ever” or “Occasionally,” or another 25.8% indicated that teaching staff recommends online videos “Sometimes.” Only 9.6% (*n* = 36) responded “Frequently” or “Almost always.” Consistent with the previous statement, students from Greece were more likely to indicate that they receive this information during classes frequently or almost always (22.4%), compared to no more than 7.8% in the other schools (OR = 2.86; 95% CI: 1.60–5.11).

When using smartphones, dental content is accessed via Google (72.2%) and YouTube (55.9%) searching, but University sites (43.7%) and PubMed (43.1%) were also well cited. Google and YouTube access via smartphones were higher than Wikipedia (28.9%), Google Scholar (21.7%) and Science Direct (7.2%) (*p* < .05). In Cardiff, the University website was students' first option to find content via mobile; in Brescia, it was Wikipedia.

Dental students (95.1%) already access dental curriculum content from the Internet. Videos are the main source of information (78.2%; *p* < .05), but step‐by‐step pictures (56.2%) were cited more (*p* < .05) than graphical illustrations (28.4%) or animations (27.6%).

YouTube videos were described as the main source of dental videos (79.9%) over their own dental school website or virtual learning environment (VLE) (49.5%). But their own dental school websites or VLEs are a preferred source of video content than other dental schools’ websites or VLEs (30.4%) or their classmates (15.2%). No differences were observed amongst the dental courses (Table 1). Most students were not aware (54.7%) of the existence of an official YouTube channel from their dental school.

### 3.2 Watching online content

Dental students have frequently (68.3%) learnt clinical procedures from online videos. As expected, students enrolled in the last years of the dental course have more chances of having seen clinical procedures from online videos than students from the 1st or the 2nd years (*p* < .001). Furthermore, for each year of dental education, there was a significant increase of 73% in the use of online videos for each year of education (OR = 1.73; 95% CI: 1.46–2.04).

Participants indicated that it would be important to watch online dental content before performing the procedure for the first time (74.8%); to see what was explained in class (65.9%); to remember how to perform clinical procedures (64.7%); to see what was read in the books (59.5%) and to visualise the procedures that they rarely see (49.8%). Eighty‐five (17.6%) students looked at videos after performing a procedure for the first time.

For 42.2% of the participants, online videos can be used to learn “Some of the procedures,” 28.7% believe that it can be used to learn “Most of the procedures,” and 11.1% of the students considered that videos can be used to learn “Few procedures.” Another 18.0% understood that videos are valid to learn “All the procedures,” ranging from none in Brescia to 39.5% in Sao Paulo (*p* < .001). However, there were no statistically significant differences by year of education. Nonetheless, students who have already learnt clinical procedures from videos are twice as likely to use videos to learn procedures than students who have not accessed videos (*p* < .01; OR = 2.27; 95% CI: 1.27–4.06; Table 1).

Students indicated the ideal video length is ranging between 5 and 15 min (53.6%) and 1 and 5 min (36.2%) (Table 1). No statistical differences were observed amongst the dental courses.

Students also mentioned that the more important video features were voice‐over (70.2%), timestamps (64.3%) and legends (51%). Music was considered the least important along with links for additional content. Athens, Sao Paulo, Brescia and Melbourne respondents considered timestamps as important as voice‐over. French respondents considered legends (subtitles) as the most important video feature.

Participants overwhelmingly (97.7%) considered that it would be useful if dental procedure videos were offered on the dental school website. Despite this, students would like to see more dental educational video content on the dental course website mainly from clinical (85.5%), pre‐clinical (74.7%) and basic sciences (50.3%; Table 1). Students also considered that educational videos work well with all the dental curricula. Particularly, with clinical (67.0%) or preclinical disciplines (61.9%) than Basic Sciences (45.4%).

Around 65% of the participants have already shared online dental videos with their colleagues. In contrast, only around one quarter (23.3%) of students discussed or showed dental educational videos found online with their teachers (Table 1). Students who learnt clinical procedures from online videos are almost three times more likely to share them with classmates (OR = 2.92; 95% CI: 1.95–4.38). However, this was not the case with sharing content with teaching staff. Nonetheless, 30.9% of those who shared content with students shared this content with their teaching staff.

Few participants were “not very confident” or “not confident at all” of online contents’ accuracy (10.4%) or about its relevance for the dental course (8.2%).

Nonetheless, the quality of online dental education materials was described as “Average” (44.2%) or “Good” or “Very Good” (42.3%). Still, the majority (77.9%) would like to receive information about how to check the reliability of online content (Table 1). As expected, for each year of education, the likelihood to be willing to receive information training on how to check the content of the source increased by 50% (OR = 1.50; 95% CI: 1.24–1.82).

Additionally, participants stated that if they face contradictory online content, they would discuss it with a classmate (40.8%), show it to one of their teachers (38.2%), ignore the resource (16.6%) or do nothing (4.4%). Although Brazilian students more frequently indicated discussing contradictions with teaching staff (48.1%), this difference was only significant for students from Melbourne who were less likely to discuss contradictory contents with teaching staff compared to students from other countries (OR = 0.33; 95% CI: 0.19–0.59). Additionally, consulting fellow students about contradictory information increased by 20% for each year of education (OR = 1.21; 95% CI: 1.06–1.37). However, this is not the case for consulting teaching staff.

## 4 DISCUSSION

In this study, the international panel of students who participated had similar use of the Internet as an extracurricular learning tool, despite their backgrounds and location. Although there were some differences by dental school location, it was not possible to identify any distinctive pattern by locality. Still, this is an empirical question with practical implications, worth to be answered. However, by years of education, there were significant differences. As expected, those in the final years were more likely to be frequent users of online resources.

Previous studies found that dental, medical and nursing students use a range of digital learning objects as non‐official learning material.^24^ Present findings would indicate that even during the so‐called “normal times” (i.e. before the COVID‐19 pandemic), the large majority of students across dental schools were using online resources as a complementary learning tool. Participants mostly used it as a “hidden curriculum” when they have doubts or as a source for additional content. Online content was also used when studying for the assessments.

Educational video platforms are appealing to students as they are more convenient and faster to access than having to check and read printed material.^35^ YouTube videos are considered as “How‐to” learning tools.^20^ Google, but not Google Scholar, and YouTube were the first and second sites more frequently reported as accessed dental content sites. Less than half reported University sites. This may be due to the fact that undergraduate dental courses do not normally offer^35^ enough open online video content to satisfy student demand,^9^ or the lack of guidance to search for content pushes students onto YouTube and Google, where sources are unreliable^35^ and the quality of their content unchecked.^36^, ^37^ This is interesting as students seemed to value their lecturers' and demonstrators' guide for online material over their peers' suggestions. However, their peers are always the first option to share and discuss what was viewed on dental videos. Only 23.4% of the students reported that they would discuss their online dental browsing habits with teachers.

This is somewhat concerning as this online material is not regulated. Reasons for this need to be further explored, but it could be because this sharing of consultation is not incentivised. In fact, most respondents indicated that rarely teaching staff recommended sites and that they learned about these resources just by searching on the Internet, not teaching staff. Alternatively, students may feel insecure to share non‐official information. Still, reported access to online resources was frequent. Moreover, a report based on recent PISA results reports difficulties in understanding and interaction with reading material online with the recent generation of students.^13^

Most students, in particular, those in the final years of their course, reported accessing clinical procedures. Thus, more chance they will learn clinical procedures online. However, according to the present results, lecturers and demonstrators do not usually recommend videos as supplementary content and most are not made aware by their students about their access to such content in their personal learning time.

The importance of training and encouraging dental students to find reliable content and to discuss fake news during the dental course has been previously raised.^38^ Dental students are not confident about the accuracy of online content but still access it due to their continuing desire to learn more about dentistry in their own way. The question remains as to how to develop the students’ abilities to review and appraise such non‐peer‐reviewed material into their clinical actions.^35^

In line with the literature,^24^ students' preference was for video content. Dental students participating in this study found the availability of the Internet on their smartphones had a positive impact on their dental academic experience. Still, the laptop computer is the preferred device when studying dental content.

### 4.1 Ways forward

Dental schools should be ready to create and publish more online video format content for their students using short videos (5–15 min) with narration, legends and timestamps. Timestamps organised according to subjects will increase students' engagement in your video and will give teachers the chance of knowing what their students’ difficulties are.^8^, ^39^, ^40^, ^41^, ^42^ Accepting that students will access the Internet for content, dental schools should provide details on how to critically appraise and incorporate online teaching materials into their learning needs and differentiate facts and evidence‐based information from just opinions or non‐evidence‐based content^13^

As in any study, there were some limitations in conducting this international research, which needs to be taken into account when evaluating these results. For example, this study used self‐reported data. In addition, the number of respondents varied, making generalisations and some comparisons difficult to make. The sample size represented a low response rate. Response rates to online surveys about oral health are within the range of 2.5%–26%.^43^, ^44^ Additionally, this was a multicentric effort and the results were impacted by local practices of data collection. Other practicalities also moderated data collection. For example, EU General Data Protection Regulation went into effect during the research delaying or preventing part of the students from receiving the survey. Brazilian Internet regulations, for instance, were not in place at that time. Other schools (e.g. Chile) were under students’ industrial actions, which also affected response rates. Australian students used a Qualtrics version of the survey; Europeans had access via email whilst Brazilians used WhatsApp. It was presumed that mobile devices (i.e. smartphones) would be the major technology vehicle to access educational and learning material, but the study did show that laptop computers were popular, and this should be considered in future surveys.^45^, ^46^

Data were collected just before the COVID‐19 outbreak. The global pandemic changes traditional learning methods. The influences and impact of measures to mitigate the pandemic effect on dental education and learning remain unknown. This emphasises the importance of understanding this impact and how students may have shifted their learning to online learning.^47^

## 5 CONCLUSIONS

Results from this assessment suggest that students are learning unofficial dental content from online videos, which include a range of clinical procedures. Overall, students from the dental schools included in the study seem to be hesitant to inform their teachers about their viewing habits but do regularly discuss the material in their peer groups. Dental students wish to receive training on how to evaluate the reliability of online content and incorporate it into their studies. Interestingly, these responses were quite consistent across the dental schools included.

## CONFLICT OF INTEREST

The authors confirm that there are no known conflicts of interest associated with this publication.

## Data Availability

The data that support the findings of this study are available on request from the corresponding author. The data are not publicly available due to privacy or ethical restrictions.

## References

[1] Khatoon B , Hill KB , Walmsley AD . Dental students' uptake of mobile technologies. Br Dent J. 2014;216(12):669‐673. 24970518 10.1038/sj.bdj.2014.523

[2] Marker DR , Juluru K , Long C , Magid D . Strategic improvements for gross anatomy web‐based teaching. Anat Res Int. 2012;2012:1‐9. 10.1155/2012/146262 PMC3335582 22567306

[3] Rajab LD , Baqain ZH . Journal of dental education. J Dent Educ. 2005;66(3):393‐404.

[4] Murakami S , Kawada E . Development and status of e‐Learning program at Tokyo Dental College. Bull Tokyo Dent Coll. 2010;51(3):119‐128. 20877158 10.2209/tdcpublication.51.119

[5] Graber ML , Tompkins D , Holland JJ . Resources medical students use to derive a differential diagnosis. Med Teach. 2009;31(6):522‐527. Available from: http://www.ncbi.nlm.nih.gov/pubmed/19811168 19811168 10.1080/01421590802167436

[6] Ahmad M , Sleiman NH , Thomas M , Kashani N , Ditmyer MM . Use of high‐definition audiovisual technology in a gross anatomy laboratory: effect on dental students’ learning outcomes and satisfaction. J Dent Educ. 2016;80(2):128‐132. Available from: http://www.jdentaled.org/content/jde/80/2/128.full.pdf 26834129

[7] Hughes JK , Kearney P . Impact of an iDevice application on student learning in an occupational therapy kinesiology course. mHealth. 2017;3:43. Available from: http://www.ncbi.nlm.nih.gov/pubmed/29184895 29184895 10.21037/mhealth.2017.08.02 PMC5682363

[8] Dias da Silva MA , Walmsley AD . Fake news and dental education. Br Dent J. 2019;226(6):397‐399. 30903059 10.1038/s41415-019-0079-z

[9] Dias da Silva MA , Pereira AC , Walmsley AD . The availability of open‐access videos offered by dental schools. Eur J Dent Educ. 2019;23(4):522‐526. 31429507 10.1111/eje.12461

[10] Infodemic [Internet] . [cited 2021 Jul 10]. Available from: https://www.who.int/health‐topics/infodemic#tab=tab_1

[11] Factsheet N . Understanding the infodemic and misinformation in the fight against COVID‐19 Department of Evidence and Intelligence for Action in Health [Internet]. [cited 2021 Jul 5]. Available from: www.paho.org/ish

[12] Greenhalgh T . Computer assisted learning in undergraduate medical education. BMJ. 2001;322(7277):40‐44. Available from: http://www.bmj.com/cgi/doi/ 10.1136/bmj.322.7277.40 11141156 PMC1119309

[13] OECD iLibrary | 21st‐Century Readers: Developing Literacy Skills in a Digital World [Internet]. [cited 2021 Jul 5]. Available from: https://www.oecd‐ilibrary.org/education/21st‐century‐readers_a83d84cb‐en

[14] Kamvar ZN , López‐Uribe MM , Coughlan S , Grünwald NJ , Lapp H , Manel S . Developing educational resources for population genetics in R: an open and collaborative approach. Mol Ecol Resour. 2017;17(1):120‐128. 27297607 10.1111/1755-0998.12558

[15] Back SJ , Darge K , Bedoya MA , et al. Ultrasound tutorials in under 10 minutes: experience and results. Am J Roentgenol. 2016;207(3):653‐660. 27276225 10.2214/AJR.16.16402

[16] Lehmann R , Seitz A , Bosse HM , Lutz T , Huwendiek S . Student perceptions of a video‐based blended learning approach for improving pediatric physical examination skills. Ann Anat. 2016;1(208):179‐182. 10.1016/j.aanat.2016.05.009 27328405

[17] Löw S , Erne H , Schütz A , Eingartner C , Spies CK . The required minimum length of video sequences for obtaining a reliable interobserver diagnosis in wrist arthroscopies. Arch Orthop Trauma Surg. 2015;135(12):1771‐1777. 26423659 10.1007/s00402-015-2339-y

[18] Stracquadanio G , Yang K , Boeke JD , Bader JS . BioPartsDB: a synthetic biology workflow web‐application for education and research. Bioinformatics. 2016;32(22):3519‐3521. 27412090 10.1093/bioinformatics/btw394 PMC5181553

[19] Guze PA . Using technology to meet the challenges of medical education. Trans Am Clin Climatol Assoc. 2015;126:260‐270. Available from: http://www.ncbi.nlm.nih.gov/pubmed/26330687 26330687 PMC4530721

[20] Smith BYA , Toor S , van Kessel P . Many Turn to YouTube for Children’s Content, News, How‐To Lessons. 2018 [cited 2020 Jul 29]; Available from: https://www.pewresearch.org/internet/2018/11/07/many‐turn‐to‐youtube‐for‐childrens‐content‐news‐how‐to‐lessons/

[21] YouTube for Press [Internet]. [cited 2021 Dec 28]. Available from: https://blog.youtube/press/

[22] Oi H , Li Y , Bailey A , et al. YouTube as a source of information on COVID‐19: a pandemic of misinformation? BMJ Global Health. 2020;5(5):e002604. Available from: https://gh.bmj.com/content/5/5/e002604 10.1136/bmjgh-2020-002604 PMC7228483 32409327

[23] Alexa Top 500 Global Sites [Internet]. [cited 2019 Feb 7]. Available from: https://www.alexa.com/topsites

[24] Li TY , Gao X , Wong K , Tse CSK , Chan YY . Learning clinical procedures through internet digital objects: experience of undergraduate students across clinical faculties. JMIR Med Educ. 2015;1(1):e1. Available from: http://www.ncbi.nlm.nih.gov/pubmed/27731303 27731303 10.2196/mededu.3866 PMC5041352

[25] Johnston ANB , Barton MJ , Williams‐Pritchard GA , Todorovic M . Youtube for millennial nursing students; using internet technology to support student engagement with bioscience. Nurse Educ Pract. 2018;31:151‐155. Available from: https://www.sciencedirect.com/science/article/pii/S1471595317308934?via%3Dihub 29906632 10.1016/j.nepr.2018.06.002

[26] Dantas AK , Shinagawa A , Deboni MCZ . Assessment of preclinical learning on oral surgery using three instructional strategies. J Dent Educ. 2010;74(11):1230‐1236. [cited 2020 Jun 10 Available from: https://pubmed.ncbi.nlm.nih.gov/21045228/ 21045228

[27] Botelho MG . The communal consultation video—enhancing learning and broadening experience through observing dialogue. Eur J Dent Educ. 2019;23(1):14‐19. 29904990 10.1111/eje.12371

[28] Nikzad S , Azari A , Mahgoli H , Akhoundi N . Effect of a procedural video CD and study guide on the practical fixed prosthodontic performance of Iranian dental students. J Dent Educ. 2012;76(3):354‐359. [cited 2020 Jun 10 Available from: https://pubmed.ncbi.nlm.nih.gov/22383605/ 22383605

[29] Marti AM , Harris BT , Metz MJ , et al. Comparison of digital scanning and polyvinyl siloxane impression techniques by dental students: instructional efficiency and attitudes towards technology. Eur J Dent Educ. 2017;21(3):200‐205. 26960967 10.1111/eje.12201

[30] Gupta B , White DA , Walmsley AD . The attitudes of undergraduate students and staff to the use of electronic learning. Br Dent J. 2004;196(8):487‐492. 15105865 10.1038/sj.bdj.4811179

[31] Walmsley AD , White DA , Eynon R , Somerfield L . The use of the Internet within a dental school. Eur J Dent Educ. 2003;7(1):27‐33. 12542686 10.1034/j.1600-0579.2003.00268.x

[32] jamovi ‐ Stats. Open. Now. [Internet]. Jamovi. 2021 [cited 2021 Jul 10]. Available from: https://www.jamovi.org/

[33] Bioestat [Internet] . Biosestat. 2021 [cited 2021 Jul 10]. Available from: https://www.mamiraua.org.br/downloads/programas/

[34] SPSS Statistics | IBM [Internet]. [cited 2021 Oct 14]. Available from: https://www.ibm.com/products/spss‐statistics

[35] Health Information National Trends Survey . In the last 12 months, have you used the Internet for any of the following reasons? Watched a health‐related video on YouTube? | HINTS [Internet]. HINTS 5 Cycle 3. 2019 [cited 2020 Nov 18]. Available from: https://hints.cancer.gov/view‐questions‐topics/question‐details.aspx?qid=1352

[36] Dias da Silva MA , Pereira AC , Walmsley AD . Who is providing dental education content via YouTube? Br Dent J. 2019;226(6):437‐440. 30903071 10.1038/s41415-019-0046-8

[37] Lena Y , Dindaroglu F . Lingual orthodontic treatment: A YouTubee video analysis. Angle Orthodont. 2018;88(2):208‐214. Available from: https://pubmed.ncbi.nlm.nih.gov/29257704/ 29257704 10.2319/090717-602.1 PMC8312536

[38] Abukaraky A , Hamdan AA , Ameera MN , Nasief M , Hassona Y . Quality of YouTube TM videos on dental implants. Med Oral Patol Oral Cir Bucal. 2018;23(4):e463–8. Available from: https://pubmed.ncbi.nlm.nih.gov/29924766/ 29924766 10.4317/medoral.22447 PMC6051691

[39] How to Add Chapters to Your Videos Using Timestamps ‐ YouTube [Internet]. YouTube Creators. 2020 [cited 2021 Jul 5]. Available from: https://www.youtube.com/watch?v=b1Fo_M_tj6w

[40] How to Make the Most of Video Timestamp Results in Google Search [Internet]. Brodie Clark. 2020 [cited 2021 Jul 5]. Available from: https://www.searchenginejournal.com/video‐timestamp‐results‐google‐search/364020/#close

[41] Improve Viewer Engagement: A Complete Guide to YouTube Timestamps [Internet]. Anthony Najera. 2021 [cited 2021 Jul 5]. Available from: https://www.shutterstock.com/blog/youtube‐timestamps‐guide

[42] Write smart descriptions ‐ YouTube Creator Academy [Internet]. YouTube Creators. 2016 [cited 2021 Jul 5]. Available from: https://creatoracademy.youtube.com/page/lesson/descriptions

[43] Goodchild J , Donaldson M . The use of sedation in the dental outpatient setting: a web‐based survey of dentists ‐ PubMed. Dent Implantol Update. 2011;22:73‐80. Available from: https://pubmed.ncbi.nlm.nih.gov/22117496/ 22117496

[44] Henry RK , Molnar A , Henry JC . A Survey of US Dental Practices’ use of Social Media. J Contemp Dent Pract. 2012;13(2):137‐141. Available from: https://pubmed.ncbi.nlm.nih.gov/22665737/ 22665737 10.5005/jp-journals-10024-1109

[45] Moraes RR , Correa MB , Daneris Â , et al. Email Vs. Instagram recruitment strategies for online survey research. Brazilian Dental Journal. 2021;32(1):67‐77. Available from: https://pubmed.ncbi.nlm.nih.gov/33914005/ 10.1590/0103-6440202104291 33914005

[46] Johnston S , Hogg W , Wong ST , Burge F , Peterson S . Differences in mode preferences, response rates, and mode effect between automated email and phone survey systems for patients of primary care practices: Cross‐sectional study. J Med Internet Res. 2021;23(1):e21240. 33427675 10.2196/21240 PMC7834947

[47] Gedera D , Brown C , Forbes D , Hartnett M , Datt A . Beyond Zoom, Teams and video lectures — what do university students really want from online learning? [Internet]. theconversation.com. 2021 [cited 2021 Oct 14]. Available from: https://theconversation.com/beyond‐zoom‐teams‐and‐video‐lectures‐what‐do‐university‐students‐really‐want‐from‐online‐learning‐167705

